# Malnutrition is associated with dynamic physical performance

**DOI:** 10.1007/s40520-019-01295-3

**Published:** 2019-08-19

**Authors:** Keenan A. Ramsey, Carel G. M. Meskers, Marijke C. Trappenburg, Sjors Verlaan, Esmee M. Reijnierse, Anna C. Whittaker, Andrea B. Maier

**Affiliations:** 1 Department of Human Movement Sciences, @AgeAmsterdam, Amsterdam Movement Sciences, Vrije Universiteit Amsterdam, Amsterdam, The Netherlands; 2 Department of Rehabilitation Medicine, Amsterdam Movement Sciences, VU University Medical Center, Amsterdam UMC, Amsterdam, The Netherlands; 3 Department of Internal Medicine, Section of Gerontology and Geriatrics, VU University Medical Center, Amsterdam UMC, Amsterdam, The Netherlands; 4 Department of Internal Medicine, Amstelland Hospital, Amstelveen, The Netherlands; 5 Department of Medicine and Aged Care, @AgeMelbourne, The Royal Melbourne Hospital, University of Melbourne, Melbourne Health, City Campus, Level 6 North, 300 Grattan Street, Parkville, VIC 3050 Australia; 6 School of Sport, Exercise and Rehabilitation Sciences, University of Birmingham, Birmingham, UK

**Keywords:** Malnutrition, Physical performance, Community dwelling, Aged, Older adults

## Abstract

**Background:**

Malnutrition and poor physical performance are both conditions that increase in prevalence with age; however, their interrelation in a clinically relevant population has not been thoroughly studied.

**Aims:**

This study aimed to determine the strength of the association between malnutrition and measures of both static and dynamic physical performance in a cohort of geriatric outpatients.

**Methods:**

This cross-sectional study included 286 older adults (mean age 81.8, SD 7.2 years, and 40.6% male) who were referred to geriatric outpatient mobility clinics. The presence of malnutrition was determined using the Short Nutritional Assessment Questionnaire (SNAQ, cut-off ≥ 2 points). Measures of dynamic physical performance included timed up and go (TUG), 4-m walk test, and chair stand test (CST). Static performance encompassed balance tests and hand grip strength (HGS). Physical performance was standardized into sex-specific Z-scores. The association between malnutrition and each individual measure of physical performance was assessed using linear regression analysis.

**Results:**

19.9% of the cohort was identified as malnourished. Malnutrition was most strongly associated with CST and gait speed; less strong but significant associations were found between malnutrition and TUG. There was no significant association between malnutrition and HGS or balance.

**Discussion:**

Physical performance was associated with malnutrition, specifically, dynamic rather than static measures. This may reflect muscle power being more impacted by nutritional status than muscle strength; however, this needs to be further addressed.

**Conclusions:**

Malnutrition is associated with dynamic physical performance in geriatric outpatients, which should inform diagnosis and treatment/prevention strategies.

## Introduction

Malnutrition and poor physical performance are two highly prevalent conditions in older adults that are associated with poor health outcomes, such as higher morbidity, mortality, and lower quality of life [1–4]. Both conditions may be highly interrelated and may potentiate each other.

Muscle is a key linking substrate between malnutrition and physical performance. Decreased muscle mass has been found to be an outcome of malnutrition [5] and decreased muscle power has been found to be a predictor of physical performance [6]. A recent meta-analysis [7] found evidence for a positive association between malnutrition and physical frailty in community dwelling adults. Physical frailty, as defined by Fried et al. [8], is a composite measure including two physical performance measures. The associations between malnutrition and individual measures of physical performance may be important to understand.

Malnutrition has been defined “a state resulting from lack of uptake or intake of nutrition that leads to altered body composition (decreased fat free mass) and body cell mass leading to diminished physical and mental function and impaired clinical outcome from disease” and, in this definition, is synonymous with undernutrition [9]. In a chronic malnourished state, adipose tissue is used as the body’s primary energy source in efforts to maximize muscle preservation [10]. However, amino acids from muscle can be required to provide 10% of energy needs for glycolytic tissues and the brain, which leads to muscle protein catabolism [11, 12]. Malnutrition is marked by muscle atrophy and overall decline in body muscle mass [10, 11]. Skeletal muscle constitutes the majority of protein-rich lean body mass and its atrophy may impede its functions including muscle strength [13], which is demonstrated by hand grip strength (HGS) and muscle power [6], which may be more reflected by dynamic physical performance tests: chair stand test (CST), gait speed, and timed up and go (TUG). Major organs, such as the heart and lungs, are not spared from being sources of energy and broken down by muscle protein catabolism, which can negatively impact cardiovascular fitness [11, 14, 15] and would likely diminish the capacity for dynamic physical performance. However, preservation of the brain may fuel neural compensation in performing cognitive aspects of physical performance tasks, such as maintaining standing balance and turning around in TUG, and limit the negative effects of malnutrition in performing these tests.

This cross-sectional inception cohort study aimed to determine the strength of association between common clinically used measures of malnutrition and measures reflecting both dynamic and static physical performance in a population of geriatric outpatients referred to a mobility clinic.

## Methods

### Study design

Older adults from two Dutch inception cohorts were included: the Bronovo cohort and the Center of Geriatrics Amsterdam (COGA) cohort. The Bronovo cohort included community-dwelling older adults referred to the geriatric outpatient mobility clinic (The Hague, The Netherlands) from March 2011 to January 2012. The COGA cohort included patients referred to the geriatric outpatient mobility clinic (Amsterdam, The Netherlands) from January 2014 to December 2015. Individuals were included if they completed a nutritional assessment questionnaire (Short Nutritional Assessment Questionnaire or Mini Nutritional Assessment) and completed at least one out of the five continuously measured physical performance tests (4-m walk test, CST, SPPB, TUG, or HGS). All patients underwent a comprehensive geriatric assessment (CGA). Patient characteristics were derived by questionnaires or obtained from medical records. Questionnaires and measurements were performed as part of CGA within routine care. The protocol and study design of the Bronovo cohort [16] and the COGA cohort [17] have been described extensively elsewhere.

Each study has been approved by the local ethical committees and has been performed in accordance with the ethical standards laid down in the 1964 Declaration of Helsinki. The need for individual informed consent was waived as the research was based on regular patient care.

### Measures of malnutrition

The presence of malnutrition was determined in the Bronovo cohort using the Short Nutritional Assessment Questionnaire (SNAQ) score, including questions about unintentional weight loss, decrease in appetite, and the use of supplemental drinks or tube feeding over the last month and has been further described elsewhere [18]. SNAQ scores range from 0 to 7 points with a score of 0–1 points is indicative of adequate nutrition; a score of 2 points indicating moderate risk of malnutrition; and a score ≥ 3 points indicating severe risk of malnutrition. In the COGA cohort, the Mini Nutritional Assessment (MNA) [19] was used to screen for malnutrition. The MNA contains questions that can be used to calculate an adapted SNAQ score, by adding the points from the first two questions pertaining to unintentional weight loss and decreased appetite, which are similar amongst the two screening tools and excluding the question pertaining to supplemental drinks or tube feeding. This calculation was done in the COGA to convert the MNA to an adapted SNAQ score with the same cut-off values as above mentioned being applied to define malnutrition. A cut-off score of 2 points or greater was used to determine the presence or absence of malnutrition from the SNAQ score or the adapted SNAQ score.

### Physical performance measures

Measures of dynamic physical performance included 4-m walk test from which gait speed can be obtained and chair stand test (CST), as sub-tests of the Short Physical Performance Battery (SPPB) [20] as well as the timed up and go (TUG). The TUG is a dynamic test where participants are asked to stand up from a seated position without using their hands, walk to a cone 3 m away, walk around the cone, walk 3 m back, and return to the sitting position without using their hands, as quickly as possible. The amount of time needed to complete all of these tasks was measured in seconds [21].

Measures of static physical performance encompassed the third subtest of the SPPB, i.e., balance tests (side by side, semi-tandem, and tandem) [20] as well as hand grip strength (HGS). HGS represents an individual’s ability to squeeze a handheld dynamometer as hard as possible with each hand three times. The maximal HGS was recorded in kilograms (kg) and used for analysis [22].

### Statistical analysis

Descriptive statistics for continuous variables were presented as mean and standard deviation (SD) when data were distributed normally or as median interquartile range [IQR] if the data had a skewed distribution. Dichotomous variables were reported by the sample size/number (*n*) and the percentage (%).

Prevalence of malnutrition according to the SNAQ score and the adapted SNAQ score were calculated in the Bronovo and COGA cohort, respectively, to test the validity of the adapted SNAQ score and provide evidence that the removal of the “supplemental drinks or tube feeding” question would not have an impact on the results.

For each measure of physical performance, respectively, individuals were included if they were able to perform the test. Continuous physical performance measures were standardized into sex-specific Z-scores to allow for direct comparison of effect sizes of malnutrition with physical performance tests. Variables with a skewed distribution were log transformed prior to making Z-scores. Linear regression analysis was used to study the association between malnutrition with both dynamic and static measures of physical performance. Balance tests were dichotomized into two groups, unable to maintain for 10 s and able to maintain for 10 s, and were included in the analysis to determine the presence of an association using a binary logistic regression. Results for the linear regression analysis are presented as beta, or for the logistic regression as odds ratios (OR), and 95% confidence intervals with *p* values. Analyses were performed unadjusted (crude model) and adjusted for age, sex, and the presence of multimorbidity. Interpretation of the results of this linear regression analysis with standardized outcome measures was as follows: the effect size represents the average difference in effect of the presence of malnutrition on the physical performance test compared to the absence of malnutrition in standard deviations.

All statistical analyses were conducted using SPSS (Statistical Package for the Social Sciences), version 24.0 (SPSS Inc. Chicago, IL, USA). A *p* value of less than 0.05 was considered statistically significant. Visualization of results was performed using GraphPad Prism 5.01.

## Results

### Participant characteristics

This study included 286 geriatric outpatients, with a mean age of 81.8 years (SD 7.2). Characteristics of the outpatients are shown in Table 1. Multimorbidity was present in 39.5% (*n* = 107) of the outpatients. Mean body mass index (BMI) was 25.4 kg/m^2^ (SD 4.3). The prevalence of malnutrition was 19.9%.

**Table 1:** Characteristics of geriatric outpatients referred to mobility clinics

Measure	Total		Bronovo		COGA	
	*n* = 286	Value	*n* = 184	Value	*n* = 102	Value
Age, mean (SD)	286	81.8 (7.4)	184	82.0 (7.3)	102	81.4 (7.6)
Male	286	116 (40.6)	184	74 (40.2)	102	42 (41.2)
Married	284	104 (36.6)	182	71 (39.0)	95	33 (34.7)
Alcohol (> 1 glass/week)	277	129 (46.6)	184	85 (46.2)	93	51 (49.5)
BMI (kg/m^2^), mean (SD)	268	25.6 (4.3)	170	25.7 (4.4)	98	25.4 (4.1)
Number of Medications, mean (SD)	273	5.8 (3.4)	179	5.3 (3.2)	94	7.0 (3.5)
Multimorbidity^a^	271	107 (39.5)	176	67 (38.1)	95	40 (42.1)
MMSE, median [IQR]	283	27.0 [25.0–29.0]	184	27.0 [24.0–29.0]	101	28.0 [26.0–29.0]
SNAQ score, median [IQR]	286	0.0 [0.0–1.0]	184	0.0 [0.0–1.0]	102	0.0 [0.0–2.0]
Unintentional weight loss	286	54 (18.8)	184	23 (12.5)	102	29 (28.4)
Decreased appetite	286	80 (27.9)	184	51 (27.7)	102	31 (30.4)
Malnutrition^b^	286	57 (19.9)	184	23 (12.5)	102	29 (28.4)
HGS (kg), mean (SD)	279	24.2 (9.0)	181	26.1 (8.4)	98	20.8 (8.9)
Male	114	31.3 (8.0)	73	33.9 (6.1)	41	26.7 (8.8)
Female	165	19.3 (5.8)	108	20.8 (5.0)	57	18.0 [13.0–20.0]
Gait speed (m/s), mean (SD)	268	0.8 (0.3)	174	0.8 (0.3)	94	0.8 (0.3)
CST time (s), median [IQR]	229	14.9 [11.7–20.0]	149	15.8 [11.6–20.1]	80	14.8 [12.1–20.0]
TUG (s), median [IQR]	235	16.1 [12.3–22.2]	160	15.8 [11.8–21.9]	75	16.5 [12.9–23.1]
SPPB score, mean (SD)	272	7.1 (3.3)	179	7.0 (3.4)	97	8.0 [5.0–10.0]
Balance: side by side^c^	272	249 (91.5)	175	161 (92.0)	97	88 (90.7)
Balance: semi-tandem^c^	271	215 (79.3)	175	143 (81.7)	96	72 (75.0)
Balance: tandem^c^	268	96 (35.8)	175	98 (56.0)	93	36 (38.7)

All variables are presented as *n* (%) unless indicated otherwise. SD, standard deviation; IQR, interquartile ratio; BMI, body mass index; SNAQ, short mini nutritional assessment questionnaire; HGS, hand grip strength; CST, chair stand test; TUG, timed up and go; SPPB, short physical performance battery

^a^Multimorbidity was defined as two or more diseases

^b^Malnutrition was determined from the SNAQ score using a cut-off of ≥ 2

^c^Balance tests were dichotomized into unable (0) and able (1) to maintain for 10 s

### Association between malnutrition and static and dynamic measures of physical performance

**Fig. 1:**
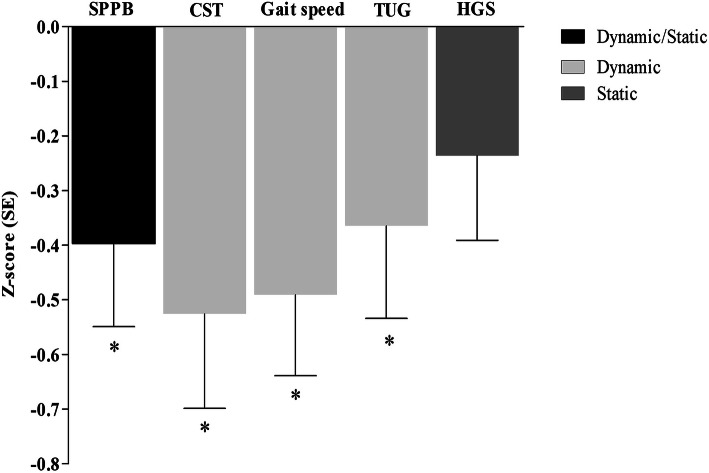
The association between malnutrition and standardized measures of physical performance stratified by type (dynamic vs. static) in geriatric outpatients adjusted for age, sex, and multimorbidity. Bars represent the difference of each physical performance test in outpatients with the presence of malnutrition compared to those without. Lower Z-score represents worse physical performance. Asterisks indicate significance at 0.05 level (* = *p* < 0.05). SE, standard error; CST, chair stand test; TUG, timed up and go; HGS, hand grip strength; SPPB, short physical performance battery

Table 2 shows the associations between malnutrition and the standardized measures of physical performance. Malnutrition associated with the composite SPPB score as well as all dynamic measures of physical performance. Malnourished patients had a 0.53 SDs longer time to complete the CST, 0.49 SDs slower gait speed, 0.37 SDs longer time to complete the TUG, and a 0.40 SDs worse score SPPB score. No statistically significant association between malnutrition and static measures of physical performance, i.e., HGS (Table 2) and balance performance (Table 3) was found. Comparing effect sizes, as shown in Fig. 1, the strongest association with the presence of malnutrition was with gait speed and, after adjustment, CST.

**Table 2:** The association between malnutrition and standardized measures of physical performance in geriatric outpatients referred to mobility clinics (*n* = 286)

	Z SPPB Score	Z LN CST	Z Gait speed	Z LN TUG	Z HGS
Crude					
*β* (95% CI)	− 0.42 (− 0.74, − 0.11)*	0.52 (0.18, 0.86)*	− 0.56 (− 0.86, − 0.25)*	0.37 (0.03, 0.72)*	− 0.27 (− 0.58, 0.04)
*p* value	0.008	0.003	0.000	0.034	0.083
Model adjusted for age, sex, and multimorbidity					
*β* (95% CI)	− 0.40 (− 0.70, − 0.10)*	0.53 (0.19, 0.87)*	− 0.49 (− 0.78, − 0.20)*	0.37 (0.03, 0.70)*	− 0.24 (− 0.54, 0.07)
*p* value	0.009	0.003	0.001	0.032	0.131

*Statistically significant results

SPPB, short physical performance battery; LN, natural log; CST, chair stand test; TUG, timed up and go; HGS, hand grip strength; *β*, beta; OR, odds ratio; CI, confidence interval

All continuous measures of physical performance were standardized and presented as sex-specific Z-scores. Variables with a skewed distribution were log transformed prior to making Z-scores

**Table 3:** The association between malnutrition and balance in geriatric outpatients referred to mobility clinics (*n* = 286)

	Balance tests^a^		
	Side by side	Semi-tandem	Tandem
Crude			
OR (95% CI)	0.66 (0.25, 1.76)	0.67 (0.33, 1.34)	0.97 (0.51, 1.80)
*p* value	0.406	0.251	0.913
Model adjusted for age, sex, and multimorbidity			
OR (95% CI)	0.69 (0.23, 2.02)	0.67 (0.31, 1.43)	1.02 (0.51, 2.04)
*p* value	0.497	0.294	0.957

OR, odds ratio; CI, confidence interval

^a^Balance tests were dichotomized into unable to maintain for 10 s (0) and able to maintain for 10 s (1)

## Discussion

In a cohort of geriatric outpatients, malnutrition as defined by SNAQ score was most strongly associated with CST and gait speed; less strong associations were found between malnutrition and TUG and the compound SPPB score. There was no significant association between HGS or balance.

Previous studies have classified physical performance into two groups: dynamic physical performance (CST, gait speed, and TUG) and static physical performance (standing balance tests and HGS) [23]. In the present study, dynamic physical performance was associated with malnutrition in contrast to static measures, which did not show a significant association. In considering the predominant physiological components of the physical performance measures, it can be argued that dynamic physical performance requires muscle power, while static physical performance (HGS) relies on muscle strength. Previous studies have shown that when assessing functional decline, it is important to acknowledge not only muscle strength (the ability to generate maximal muscle force) but also muscle power (the product of the force and velocity of muscle contraction) [6]. Muscle power has been shown to decline more rapidly with increasing age than strength, and to be a more discriminant predictor of functional performance than muscle strength [6]. As malnutrition (undernutrition) creates a negative energy balance and is associated with decreased energy expenditure [24], a malnourished individual’s ability to supply the rapid burst of energy required to exert a muscle power may be diminished. Therefore, results may provide evidence for further investigation into the impact of malnutrition on discrete measures of muscle power as the physical performance tests in the current study involve multiple systems and muscle functions.

Our study confirms the previously reported associations between malnutrition and CST, gait speed, TUG, and SPPB Score [25–32], with the added novel component of comparing the effects of the associations. We expected that malnutrition would show the strongest association with HGS as a direct measure of muscle function, i.e., strength. However, there is debate regarding the relationship between HGS and nutritional status. Some studies have advocated for the added value of HGS in nutritional assessment [33, 34], while others, including the current study, provide evidence that HGS may be of limited use as a predictor of nutritional status [35–37]. A recent study showed that HGS and knee extension strength (KES) have moderate-to-low agreement, indicating that HGS should not be used to represent overall muscle strength [38]. Furthermore malnutrition has been shown to have a greater negative effect on KES, in comparison to HGS [39]. These findings may further provide evidence that upper body and lower body strength may be impacted by nutritional status differently.

As hypothesized, none of three balance tests were associated with malnutrition. A previous study showed that muscle strength, measured by HGS and KES, rather than muscle mass, were positively associated with ability to maintain standing balance [40]. This result that balance was most dependent on muscle strength may provide additional context for the results present study as HGS, muscle strength, was also not associated malnutrition.

There is no gold standard for the diagnosis, screening, or definition of malnutrition, so the results may be highly dependent on the measure used to define malnutrition. The SNAQ, which was used in this study, adopts a broad definition of malnutrition that specifically reflects undernutrition by emphasis on unintentional weight loss and loss of appetite [9, 18, 41]. The SNAQ is often used in outpatient settings due to its practical ease, and has moderate sensitivity and high specificity in the current population with respect to diagnostic accuracy [41, 42]. However, previous studies have found the use of different nutritional screening tools which can give different prevalence estimates of malnutrition in the same cohort and different validities [43, 44]. While the unintentional weight loss and decreased appetite questions in the SNAQ and MNA are similar, they are structured differently, which may have caused a difference between SNAQ and adapted SNAQ. Furthermore, because the SNAQ screens for malnutrition rather than diagnosing it, this study uses the terms “malnutrition” and “risk of malnutrition” interchangeably, as is often the case in previous studies.

To our knowledge, this is the first study to compare the effects of the association of malnutrition with different measures of physical performance in geriatric outpatients. With the methods used in this cross-sectional study, it is difficult to distinguish the underlying systems involved in physical performance that may have been affected by malnutrition because of mutual interaction. Future studies should aim to identify these systems as well as factors that may influence the relationship between malnutrition and physical performance. There is clinical need to expand on these findings and further characterize the interrelation between the nutritional and physical state in older adults.

This study shows that physical performance measures have individual value in relation to malnutrition and should not be used on their own interchangeably to represent physical performance; the present results suggest that focusing on measures of dynamic physical performance and muscle power would be most informative in terms of reviewing the physical impact of nutritional status. Furthermore, future studies should distinguish and examine the association between malnutrition different outcomes related to muscle function, including muscle power, muscle strength, and muscle mass.

## Data Availability

The data sets generated during and/or analysed during the current study are available from the corresponding author on reasonable request.
